# Dental Pulp: Correspondences and Contradictions between Clinical and Histological Diagnosis

**DOI:** 10.1155/2015/960321

**Published:** 2015-05-11

**Authors:** Cristian Levente Giuroiu, Irina-Draga Căruntu, Ludmila Lozneanu, Anca Melian, Maria Vataman, Sorin Andrian

**Affiliations:** ^1^Department of Odontology, Periodontology, and Fixed Prosthesis, Faculty of Dental Medicine, University of Medicine and Pharmacy “Grigore T. Popa”, 16 University Street, 700115 Iaşi, Romania; ^2^Department of Morpho-Functional Sciences, University of Medicine and Pharmacy “Grigore T. Popa”, 16 University Street, 700115 Iaşi, Romania

## Abstract

Dental pulp represents a specialized connective tissue enclosed by dentin and enamel, the most highly mineralized tissues of the body. Consequently, the direct examination as well as pathological evaluation of dental pulp is difficult. Within this anatomical context, our study aimed to evaluate the correlation between dental pulp lesions and clinical diagnosis. Pulpectomies were performed for 54 patients with acute and chronic irreversible pulpitides and for 5 patients (control group) with orthodontic extractions. The morphological features were semiquantitatively assessed by specific score values. The clinical and morphological correspondence was noted for 35 cases (68.62%), whereas inconsistency was recorded for 16 cases (31.38%). The results of the statistical analysis revealed the correlations between clinically and pathologically diagnosed acute/chronic pulpitides. No significant differences were established between the score values for inflammatory infiltrate intensity, collagen depositions, calcifications and necrosis, and acute, respectively chronic pulpitides. We also obtained significant differences between acute pulpitides and inflammatory infiltrate and calcifications and between chronic pulpitides and inflammatory infiltrate, collagen deposition, and calcifications. On the basis of the predominant pathological aspects, namely, acute and chronic pulpitis, we consider that the classification schemes can be simplified by adequately reducing the number of clinical entities.

## 1. Introduction

Pulp pathology is the generic term used in the description of pulp diseases as a morphofunctional ensemble. The clinical diagnosis relies on the correlation of information regarding the inflammatory state of dental pulp described by the patient through clinical symptoms, mainly pain, which together with other paraclinical data (dental radiographies) provide the basis for a therapeutic decision [1]. Dental pulpitides represent a separate class of endodontic pathology initiated, in most cases, by a progressive dynamic process of dental caries. Acute or chronic inflammatory reactions of the pulp, occurring as a result of exposure of the pulpodentinal tissue, are caused by various invasion modalities involving or not the dentinal tubules [2–4].

The loose pulp tissue is protected by enamel and dentin, structures with a high degree of mineralization. Therefore, its direct inspection and palpation as well as the pathological assessment become difficult tasks. Nevertheless, an immediate therapeutic intervention is essential for the maintenance of pulp vitality [5, 6]. Hence, inflammatory pulp diseases are a major challenge for the endodontic specialists, requiring both practical experience and theoretical knowledge [5–7].

The mainstream publications report rather few studies focused on the relationship between the pathological and clinical status in dental pulp diseases [1, 8–14]. These studies draw the attention to the lack of concurrence between the clinical and pathological pictures, which therefore is deemed as a major impediment for the diagnosis accuracy, with a significant impact in endodontic practice [1, 10, 11, 14].

Within this framework, the purpose of our study was to investigate the morphological changes in the acute and chronic pulpodentinal inflammatory diseases in parallel with the clinical characteristics, aiming at evaluating the correlation degree between the pathological lesions and clinical diagnosis.

## 2. Material and Methods

### 2.1. Patients

The study group consisted of 59 patients (30 males and 29 females), aged between 18 and 67 years old (i.e., 38.83 mean age), registered in the interval of 2011–2014 for diagnosis and treatment at the “M. Kogălniceanu” Dentist Medical Learning Base, University of Medicine and Pharmacy “Grigore T. Popa” of Iaşi, Romania. From the 59 patients, 54 were diagnosed with acute and chronic irreversible pulpitides, on which pulpectomies were performed. The remaining 5 patients, with no dental caries or periodontal diseases, benefited from orthodontic extractions, with preceding pulpectomies; they represented the control group.

The study was approved by the Ethical Committee of the University of Medicine and Pharmacy “Grigore T. Popa” of Iaşi on the basis of the written informed consent of the patients relative to the usage of biological material harvest through pulpectomies (protocol number 10353). The research was conducted in full accordance with the World Medical Association Declaration of Helsinki.

### 2.2. Clinical Exam

The clinical diagnosis of irreversible pulpitis was achieved taking into account the following symptoms and signs: (i) spontaneous pain, (ii) positive response to the pulp sensitivity testing: prolonged pain and maximum 8 minutes after removing the cold stimulus caused by Tetrafluoroethane (Pharmaethyl Spray, Septodont), (iii) positive response to the pulp sensitivity testing: prolonged pain and maximum 8 minutes after removing the warm stimulus caused by warmed gutta-percha (gutta-percha sticks, h01061, Coltene), (iv) prolonged pain and maximum 8 minutes to sweet stimuli, (v) pain to the percussion on the tooth axis, and (vi) bleeding and pain at the palpation of the pulp tissue exposed in the oral cavity [15]. The pulp sensitivity testing was performed comparatively with the neighboring or homologous teeth.

To discriminate between acute and chronic inflammation, we used the criteria summarized by Table 1, which express our clinical expertise in pulpitides diagnosis.

### 2.3. Therapeutic Protocol of the Vital Extirpation of Pulp Tissue (Pulpectomy)

In accordance with the pulpectomy procedure [3] the following stages were performed: locoregional anesthesia (Ubistesin Forte 4%, 3M ESPE), isolation of operative field with dam and application of saliva evacuator, antisepsis of operative field with 2% sodium hypochlorite (Chloraxid 2%, Cerkamed, Poland), and preparation of the access cavity to the selected place, specific for the approached tooth. The working length was set at 1 mm from the radiologic apex detected on a digital dental radiography and compared with the length identified by the apex locator Root ZX II (J. Morita, USA). Permeation of the endodontic space was performed with a Kerr file needle (Sendoline-Poldent, Poland) in order to generate an area for the excision of the connective tissue with Tire Nerf needles (Sendoline-Poldent, Poland).

After the removal of the pulp tissue, the enlargement of the root canal (manual needles Protaper, Dentsply, USA), irrigation with antiseptic substances (2% sodium hypochlorite, Chloraxid 2%, Cerkamed, Poland, and 3% oxygenated water, Tis Farmaceutic, Romania), and definitive filling (Sealapex, Kerr Corporation, USA, and gutta-percha sticks Protaper, Dentsply, USA) were performed in the same therapeutic session.

### 2.4. Pathologic Examination

The 59 pulp specimens (corresponding to the number of patients in the study group) were immersed immediately after harvesting in 10% buffered formalin. After fixation, the specimens were processed in accordance with the standard protocol for the histopathological exam. From the paraffin blocks, 4 *μ*m seriated sections were cut and stained with hematoxylin-eosin (HE) and trichrome light green (special staining for collagen). Three specimens were lost because of technical problems (very small size of harvested pulp tissue), and thus the final investigation group consisted of 56 specimens (among which 5 specimens correspond to the control group).

### 2.5. Semiquantitative Evaluation

For each specimen, the semiquantitative evaluation was performed on at least 7 microscopic fields, analyzed at ×400 magnification. We examined the following four morphological features: (i) intensity of inflammatory infiltrate, (ii) collagen deposition, (iii) presence of pulp calcification, and (iv) necrosis. We allocated score values [3] reflecting the lesion extension relative to each feature, as shown in Table 2. A final score was calculated as a sum of the four score values.

### 2.6. Statistical Analysis

The performance of the clinical exam (compared to the pathological exam, considered as gold standard) for the diagnosis of acute and chronic pulpitis was assessed by the calculation of sensitivity and specificity.

The correlation between the clinical and pathological exam was statistically analyzed by using the Spearman *R* correlation test and Mann-Whitney test. For Spearman correlation coefficient (*r*_*S*_), the strength of correlation was assessed in accordance with the absolute value as follows: 0.00–0.19: very weak, 0.20–0.39: weak, 0.40–0.59: moderate, 0.60–0.79: strong, and 0.80–1.00: very strong. The level of significance was set to *P* values < 0.05.

## 3. Results

### 3.1. Clinical versus Pathological Diagnosis

Relying on the criteria applied for the clinical diagnosis, 19 cases (37.26%) had acute pulpitides and 32 cases (62.74%) had chronic pulpitides.

From the 19 cases clinically diagnosed as acute pulpitides, 7 cases were confirmed by the pathological exam. For the 32 cases with the clinical diagnosis of chronic pulpitides, the pathological exam confirmed 28 cases. Consequently, we obtained the clinical and morphological correspondence for 35 cases (68.62%), the inconsistency between the clinical and pathological diagnosis being recorded in 16 cases (31.38%). The discrepancy between diagnoses included 12 cases clinically diagnosed as acute pulpitides, but morphologically described as chronic pulpitides, and 4 cases clinically diagnosed as chronic pulpitides but with pathological modifications specific to the acute pulpitides.

### 3.2. Pathological Characteristics

**Figure 1:**
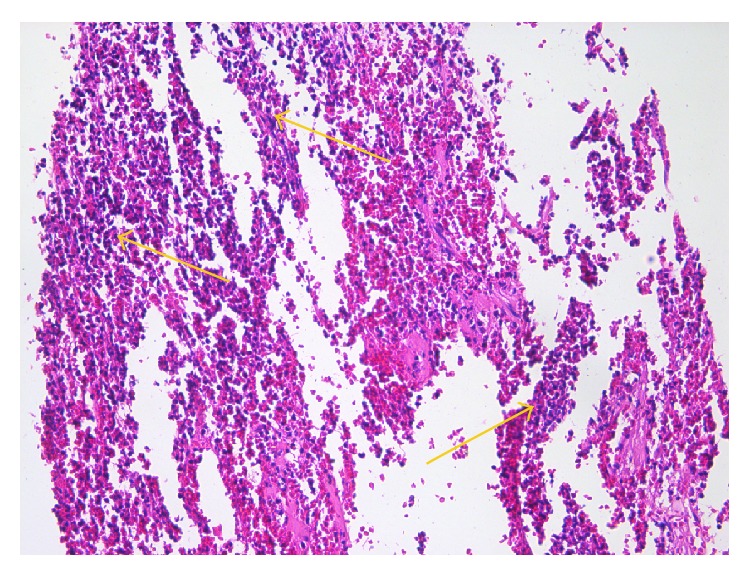
Acute pulpitis: diffuse acute inflammatory infiltrate (arrows) predominantly consisting of neutrophils (HE, ×200).

The cases diagnosed by the pathological exam as acute pulpitides presented the following alterations in the pulp tissue: vascular congestion and diffuse polymorphic inflammatory infiltrate mostly perivascular, associated with odontoblastic degeneration, interstitial matrix edema, and cellular detritus (Figure 1). The inflammatory infiltrate, integer and/or altered, together with cellular detritus was responsible for the infiltrative and destructive nature of the lesions typical to acute pulpitides.

**Figure 2:**
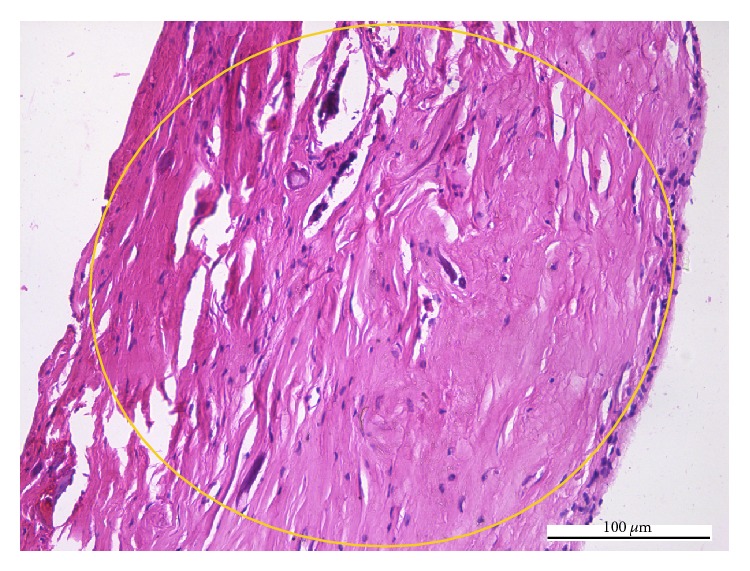
Chronic pulpitis: the collagen proliferation (circle) replaces the normal pulp tissue (HE, ×200).

**Figure 3:**
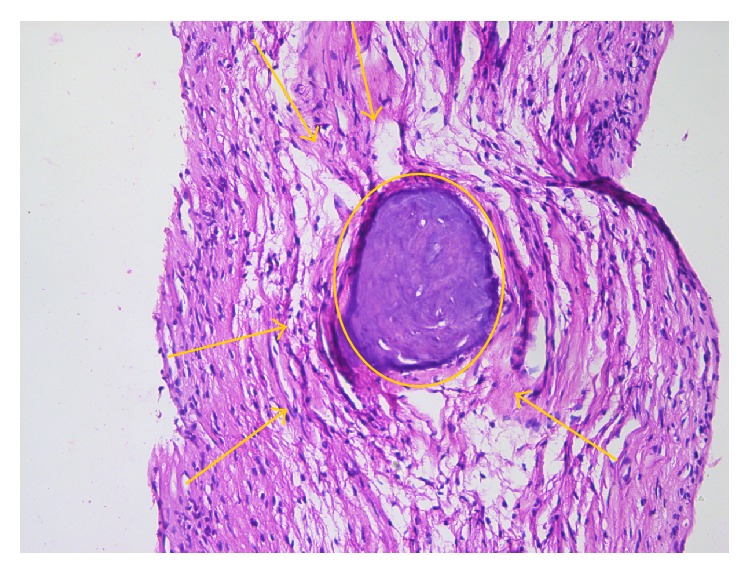
Chronic pulpitis: collagen accumulation (arrows) in central pulp area, surrounding a pulp stone (circle) (HE, ×200).

**Figure 4:**
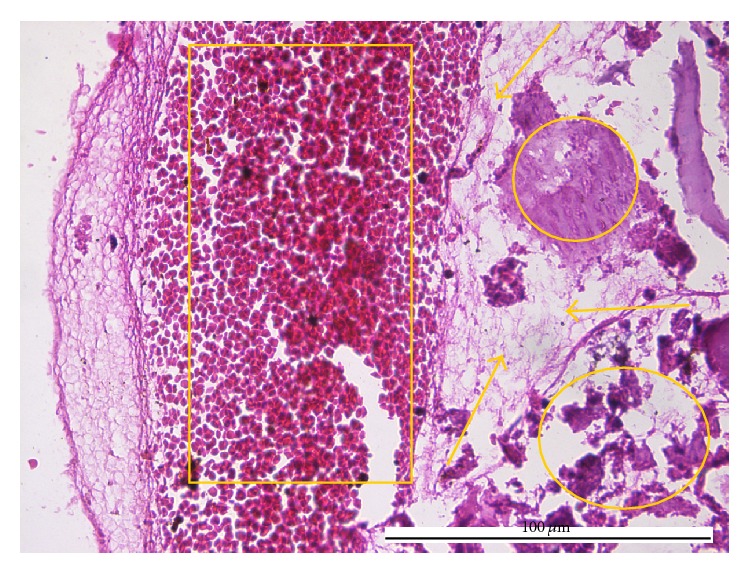
Chronic pulpitis: massive hemorrhage (box), edema (arrows), and calcification (circles) (HE, ×400).

**Figure 5:**
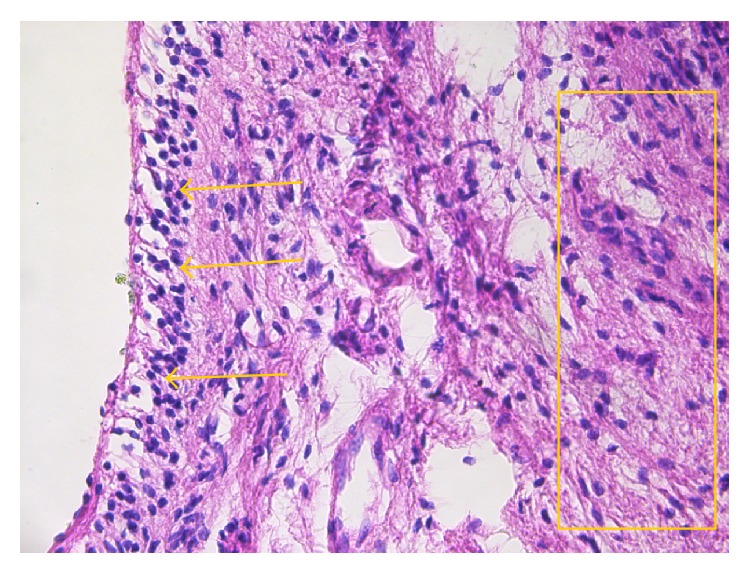
Chronic pulpitis: odontoblasts (arrows) covering the collagenized central pulp (box) (HE, ×200).

The cases pathologically diagnosed as chronic pulpitides were characterized by conspicuous collagen deposition in the central pulp (Figure 2) which progresses to fibrosis, areas with frequent fibroblasts, areas with isolated fibroblasts, chronic inflammatory infiltrate, calcifications with dentinal tubules—real pulpolites (Figure 3), diffuse calcifications with linear placement, associated with blood vessels (frequently with sclerotic walls), hemorrhages, prominent tissue edema (Figure 4), and cellular detritus. We point out that in chronic pulpitides (as opposed to the acute ones) the peripheral odontoblastic layer was preserved (Figure 5).

The semiquantitative analysis yielded for the 51 pulp specimens final scores between 1 and 6, as showed in Table 3.

The application of the scoring system for the evaluation of the severity degree of the lesions revealed similar final scores for both types of pulpitides, even if they result from the summation of different score values for one or more of the evaluated features.

The five cases belonging to the control group showed the preservation of normal morphology for the peripheral pulp (with odontogenic potential through the odontoblastic layer), the cell-free zone (zone of Weil), and cell-rich zone as well as for the loose connective tissue of central pulp, with a rich blood and nerve supply. The semiquantitative analysis applied to these specimens led to final score 0 for all cases.

### 3.3. Statistical Correlations

For acute pulpitides, the sensibility and specificity of clinical diagnosis were 36% and 87%, respectively. For chronic pulpitides, the sensibility and specificity of clinical diagnosis were 87% and 36%, respectively.

The statistical analysis based on the Spearman test revealed the presence of a significant difference between the diagnosis of acute and chronic pulpitis, established by clinical and by pathological exam (*P* = 0.045). The *r*_*S*_ absolute value (0.282) proved a weak correlation.

The application of Mann-Whitney test for the exploration of differences between the score values for the inflammatory infiltrate, collagen depositions, calcifications, and necrosis and for the acute and chronic pulpitides, respectively, yielded statistically nonsignificant results for all four features (*P* > 0.05).

Spearman test used for the correlation between each of the four features on one hand, and the pathological diagnosis of acute pulpitis on the other, proved significant differences for the intensity of inflammatory infiltrate (*P* = 0.002), with a very strong correlation (*r*_*S*_ = 0.906), and for the existence of pulp calcifications (*P* = 0.037), with a strong correlation (*r*_*S*_ = 0.736).

Spearman test also revealed statistically significant correlations between the intensity of the inflammatory infiltrate, collagen deposition, and pulp calcifications and the diagnosis of chronic pulpitis (all *P* = 0.0001). The correlation was moderate for the intensity of the inflammatory infiltrate and collagen deposition (*r*_*S*_ = 0.518 and *r*_*S*_ = 0.513, resp.) and strong for the presence of calcifications (*r*_*S*_ = 0.634).

## 4. Discussion

Although the mainstream publications largely describe pulp pathology from the clinical and therapeutic perspective, very few studies concentrate on the relationship between the clinical diagnosis and the pathological substrate specific to the inflammatory process developed in the pulp [1, 8–14].

The first attempt to classify pulp lesions was made in 1959, when Held and coworkers defined acute pulpitis as a consequence of the exacerbation of an existent chronic pulpitis, the pulp tissue being already chronically inflamed as a consequence of the evolution of caries process [16]. Similar results were also obtained by Seltzer and Bender in 1963, Hess in 1965, and Baume and coworkers in 1974, who outlined that most acute inflammations of dental pulp are not primary processes but inflammations secondary to preexistent chronic reactions turned acute [8, 17, 18]. Over the time, the interest in this subject became visible in numerous anatomoclinical classifications that take into account a full overlap between clinical symptoms and histopathological changes in the dental pulp [1]. The experience of the Romanian dentistry school took the shape of an anatomoclinical classification which comprises the following entities: (i) pulp hyperemia, (ii) partial/total serous acute pulpitis, (iii) partial/total purulent acute pulpitis, (iv) open ulcerous/polypoid chronic pulpitis, (v) hyperplastic/true closed chronic pulpitis, (vi) pulp necrosis, and (vii) pulp degenerative lesions [19].

Despite all these attempts, a clinicopathological classification unanimously accepted by the medical community (as a gold standard) does not exist and further investigations are required towards a reliable consensus able to refine the connections between clinical and pathological findings.

Our study revealed pathological modifications similar to the ones described in literature, which determined the diagnosis of acute or chronic pulpitis, respectively. Our data showed that the clinical and pathological diagnosis coincided for 35 from 51 cases, which means 68.62%. To the best of our knowledge, results of this type have been reported by the mainstream publications in a single study [10] that found a percentage of 49.54% for 109 analyzed cases. One may consider our percentage as a better value in the sense of a more accurate correspondence between clinical and pathological exams. It is worth also mentioning that a recent study [14] addresses the slightly different problem of reversible and irreversible pulpitides; the match between clinical and pathological diagnosis was 96.6% for normal pulp/reversible pulpitis and 84.4% for irreversible pulpitis.

We consider our results relevant because we provide statistical analysis arguments for the diagnosis match (approximately 70%) and diagnosis inconsistency (approximately 30%). Thus, we obtained a significant correlation (*P* = 0.045) between the diagnoses of acute and chronic pulpitis, respectively, established by clinical and pathological exam, but we found the value of 0.282 for the Spearman correlation coefficient. The statistical analysis also proves that the key feature in defining the degree of pulp lesion in acute pulpitis is the intensity of the inflammatory infiltrate (*P* = 0.002; *r*_*S*_ = 0.906), whereas the expansion of collagen depositions (*P* = 0.0001; *r*_*S*_ = 0.518) together with the inflammatory infiltrate (*P* = 0.0001; *r*_*S*_ = 0.513) mainly defines the degree of pulp lesion in chronic pulpitis. A special mention must be made for pulp calcifications, whose statistically significant presence (*P* = 0.037 and *r*_*S*_ = 0.736 in acute pulpitis; *P* = 0.0001 and *r*_*S*_ = 0.634 in chronic pulpitis) could be interpreted as the result of the permanent reparatory process in the dental pulp, damaged either by acute or chronic inflammation.

The discrepancies between the pathological diagnosis of chronic pulpitides and the clinical diagnosis of acute pulpitides (12 cases) can be explained through the mechanism of reactivation of chronic lesions [20]. In these cases, the clinical diagnosis was based on intermittent episodes of minor to moderate pain, with various irradiations. These symptoms orient the clinician towards the diagnosis of acute pulpitis but, in fact, they are due to the previous pulp modifications, most likely asymptomatic, which the patient cannot document in the anamnesis [21]. Morphologically, in the necrosis areas already existing in the pulp the proteolytic products act as secondary irritants on pulp tissue, responsible for the pain sensation [7, 8].

**Figure 6:**
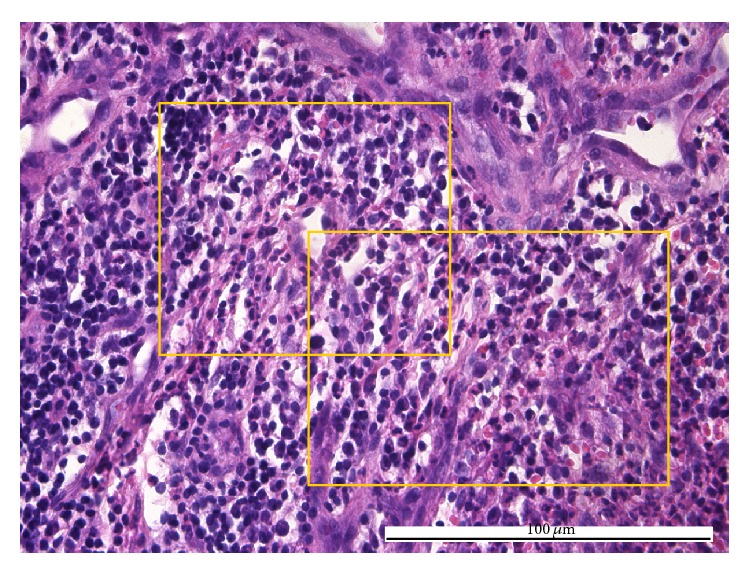
Acute pulpitis: diffuse acute inflammatory infiltrate (boxes) predominantly consisting of neutrophils with multilobate nuclei integer and/or altered eosinophils and extravasated red blood cells (HE, ×400).

**Figure 7:**
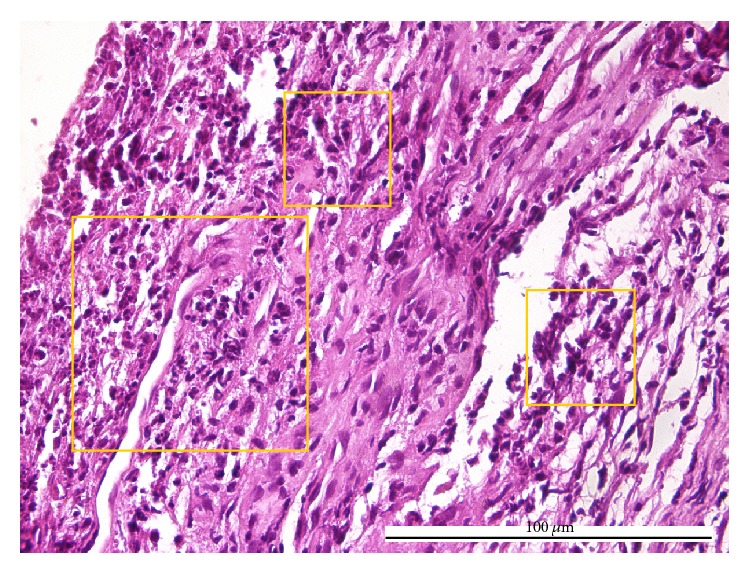
Acute pulpitis: diffuse acute inflammatory infiltrate (boxes) consisting of neutrophils and eosinophils and homogenous collagen fibers (HE, ×400).

The explanation for the reversed type of diagnosis inconsistencies (i.e., pulpitides pathologically identified as acute and clinically considered as chronic, 4 cases) may be given as follows: (i) for proper closed chronic pulpitis, by the secondary effects of a prolonged exposure to an asymptomatic carious process [6], namely, the increase of intratissular pressure, acidity, and hypoxia, followed by an afflux of acute leukocytes (Figure 6) and (ii) for open granulomatous chronic pulpitis, by the overlapping of the pulp chamber opening [20] and the commencement of the change from acute to chronic inflammation (Figure 7).

Finally, our discussions highlight the impact of the clinicopathological correlation on the endodontic practice. Thus, this study draws the attention to the difficulty to accurately establish a correct diagnosis based solely on clinical criteria. In parallel, we underline the fact that the evaluation of the pathological status of the dental pulp without correlation with the clinical context seems not to yield reliable results. Similar but isolated observations were published in the literature of the ‘60s [8, 11] and also recently [12, 14]. Unfortunately, their scientific messages were not able to influence the strategy of pulp disease classification.

In our opinion, the currently in use classifications associate clinical entities with morphological entities that are somewhat outdated. This statement, apparently bold, is strongly supported by the fact that the pathological evaluation of the study group relied on only two diagnosis entities: acute and chronic pulpitis.

In any of the 51 analyzed cases, we did not identify the specific morphological types: hyperplastic, granulomatous, polypoid, or ulcerated pulpitis. The modification of the pathological spectrum previously attributed to the pulp tissue reflected by the absence (or extremely scarce presence) of particular inflammatory subtypes could be the result of the social and economic context defining the current society: increased accessibility of specialized medical care and an increased medical education of population, including mass media means. Moreover, we could also point out the benefits of the antibiotics and analgesics administered for other systemic diseases, on the pulp tissue. Last but not least in the evaluation of the inflammatory reactivity and regeneration ability of the dental pulp, it must be taken into account the intervention of the following factors: quantity, duration and pathogenicity of bacterial biofilm [22], dental traumatic lesions [23], and decrease in pulp density due to the advanced age of the patient and to dental functional stimulation [24].

## 5. Conclusion

Our study outlines a relatively weak correlation between the clinical and pathological diagnoses for acute and chronic pulpitides. Consequently, we assume that the attention of the specialists must focus on updating the classifications for pulp pathology. On the basis of the predominant pathological aspects, namely, acute and chronic pulpitis, we propose their use only and consider the fact that the classification schemes can be simplified (by adequately reducing the number of clinical entities).

## Figures and Tables

**Table 1:** Main criteria used in differential diagnosis between acute and chronic pulpitis.

Differential diagnosis between acute and chronic pulpitis		
Criteria	Acute pulpitis	Chronic pulpitis
Dental history	First dental pain in the causal tooth which goes to dental emergencies	More episodes of dental pain caused which did not lead the patient to go at dental emergencies

Painkiller	Pain does not respond to analgesics	Pain goes to analgesics

Pain type	Intense, sharp, progressive	Dull or annoying

Onset	Suddenly, fulminatory	Insidious

Duration/time frame of occurrence	From a few hours to 24–48 hours	From several minutes to several hours (up to 2 hours)

Pain location	Irradiance, diffuse	Located

Stimulus	Heat and cold	A painful embarrassment often felt during chewing

Percussion in the tooth	Positive response	Negative response

Pulp test	Hyperexcitability at a lower intensity of thermal stimulant	Hypoexcitability at a higher intensity of thermal stimulant

Causes	Primary acute deep tooth decay or fillings adjacent but with pulp chamber closed	Primary chronic dental caries or recurrent under fillings adjacent but with pulp chamber closed or open. Affected teeth with dental erosion or vital teeth prepared for fixed prosthetic crown.

Radiograph	Coronary radiolucent areas (caused by tooth decay or erosion) or radiolucent coronal dentin under a filling but very close to the celling of pulp chamber	

**Table 2:** Meaning of the score values used in the semiquantitative evaluation.

Features	Score		
Inflammatory infiltrate	0	1	2
	Absent	Mild, <35% of specimen area	Intense, >35% of specimen area

Collagen deposition		1	2
		Mild, <35% of specimen area	Intense, >35% of specimen area

Pulp calcification	0	1	2
	Absent	Mild, <35% of specimen area	Intense, >35% of specimen area

Necrosis	0	1	
	Absent	Present	

**Table 3:** Score values and final scores for the investigated pulpitides.

Final score	Histologic features												Total number of cases
	Acute pulpitis						Chronic pulpitis						
	Number of cases		I_I	C_D	P_C	N	Number of cases		I_I	C_D	P_C	N	
**1**	**1**	1	0	1	0	0	**1**	1	0	1	0	0	**2**

**2**	**2**	1	0	2	0	0		1	1	1	0	0	**6**
		1	0	1	1	0	**4**	1	0	1	1	0	
								2	0	2	0	0	

**3**	**5**	3	0	2	1	0		1	2	1	0	0	**20**
								2	1	1	1	0	
		1	0	1	2	0	**15**	3	1	2	0	0	
		1	1	1	1	0		4	0	2	1	0	
								5	0	1	2	0	

**4**	**2**	1	1	2	1	0	**13**	2	1	1	2	0	**15**
								4	0	2	2	0	
								7	1	2	1	0	

**5**	**1**	1	1	2	2	0	**5**	5	1	2	2	0	**6**

**6**	**0**	0	—	—	—	—	**2**	1	2	2	2	0	**2**
								1	1	2	2	1	

I_I: inflammatory infiltrate, C_D: collagen deposition, P_C: pulp calcification, and N: necrosis.

## References

[1] Abbott P. V., Yu C. (2007). A clinical classification of the status of the pulp and the root canal system. *Australian Dental Journal*.

[2] Bergenholtz G. (1981). Inflammatory response of the dental pulp to bacterial irritation. *Journal of Endodontics*.

[3] Bruno K. F., Silva J. A., Silva T. A., Batista A. C., Alencar A. H. G., Estrela C. (2010). Characterization of inflammatory cell infiltrate in human dental pulpitis. *International Endodontic Journal*.

[4] Izumi T., Kobayashi I., Okamura K., Sakai H. (1995). Immunohistochemical study on the immunocompetent cells of the pulp in human non-carious and carious teeth. *Archives of Oral Biology*.

[5] Mejàre I. A., Axelsson S., Davidson T. (2012). Diagnosis of the condition of the dental pulp: a systematic review. *International Endodontic Journal*.

[6] Ward J. (2002). Vital pulp therapy in cariously exposed permanent teeth and its limitations. *Australian Endodontic Journal*.

[7] Bender I. B. (2000). Pulpal pain diagnosis—a review. *Journal of Endodontics*.

[8] Seltzer S., Bender I. B., Ziontz M. (1963). The dynamics of pulp inflammation: correlations between diagnostic data and actual histologic findings in the pulp. *Oral Surgery, Oral Medicine, Oral Pathology*.

[9] Tyldesley W. R., Mumford J. M. (1970). Dental pain and the histological condition of the pulp. *The Dental Practitioner and Dental Record*.

[10] Garfunkel A., Sela J., Ulmansky M. (1973). Dental pulp pathosis: clinicopathologic correlations based on 109 cases. *Oral Surgery, Oral Medicine, Oral Pathology*.

[11] Lundy T., Stanley H. R. (1969). Correlation of pulpal histopathology and clinical symptoms in human teeth subjected to experimental irritation. *Oral Surgery, Oral Medicine, Oral Pathology*.

[12] Cisneros-Cabello R., Segura-Egea J. J. (2005). Relationship of patient complaints and signs to histopathologic diagnosis of pulpal condition. *Australian Endodontic Journal*.

[13] Yamamoto H., Gomi H., Kozawa Y., Yamaura Y., Matsushima K., Yamazaki M. (1987). A comparative study between clinical and pathological diagnoses using extirpated pulps. *The Journal of Nihon University School of Dentistry*.

[14] Ricucci D., Loghin S., Siqueira J. F. (2014). Correlation between clinical and histologic pulp diagnoses. *Journal of Endodontics*.

[15] Bender I. B. (2000). Reversible and irreversible painful pulpitides: diagnosis and treatment. *Australian Endodontic Journal*.

[16] Schreyer C. O. (1971). Les troubles de la mastication. *Zeitschrift für Präventivmedizin*.

[17] Hess J. C. (1965). Endodontic therapeutics. *Revue Française d'Odonto-Stomatologie*.

[18] Baume L. J., Holz J., Fiore-Donno G. (1974). Clinical management of endodontic problems based on new histopathological evidence. *Die Quintessenz*.

[19] Gafar M., Andreescu C., Iliescu A. (1977). Value of anatomo-clinical diagnosis in the treatment of acute pulpitis. *Revista de Chirurgie, Oncologie, Radiologie, O. R. L., Oftalmologie, Stomatologie*.

[20] Bergenholtz G. (1990). Pathogenic mechanisms in pulpal disease. *Journal of Endodontics*.

[21] Michaelson P. L., Holland G. R. (2002). Is pulpitis painful?. *International Endodontic Journal*.

[22] Kishen A., Haapasalo M. (2010). Biofilm models and methods of biofilm assessment. *Endodontic Topics*.

[23] Raslan N., Wetzel W.-E. (2006). Exposed human pulp caused by trauma and/or caries in primary dentition: a histological evaluation. *Dental Traumatology*.

[24] Murray P. E., Stanley H. R., Matthews J. B., Sloan A. J., Smith A. J. (2002). Age-related odontometric changes of human teeth. *Oral Surgery, Oral Medicine, Oral Pathology, Oral Radiology, and Endodontics*.

